# Institutional and Cultural Barriers to ACP: Staff Perspectives

**DOI:** 10.1093/geroni/igaa057.2718

**Published:** 2020-12-16

**Authors:** Gloria Gutman, Brian de Vries, Helen Kwan, Katrina Jang, Shimae Soheilipour

**Affiliations:** 1 Simon Fraser University, Vancouver, British Columbia, Canada; 2 San Francisco State University, San Francisco, California, United States; 3 Gerontology Research Centre, Vancouver, British Columbia, Canada

## Abstract

This study explored staff knowledge and engagement in assisting residents/families with ACP. Focus groups were conducted at two long-term care homes, one Exclusively Chinese (EC; n = 25); one Multi-Ethnic (ME; n = 41). In each, separate focus groups were held with registered staff, care aides, and support staff who also completed brief surveys providing socio-demographic data and information about their training and experience with ACP. Perceived barriers to engagement in ACP included limited knowledge and inadequate training in facilitating ACP, cognitive impairment of residents, language barriers, lack of openness to discussing ACP, family expectations and misinformation. EC staff also considered cultural and religious beliefs as one of the main barriers to engaging in ACP both for residents and families; staff at ME focused more on timing and the role of family. Support staff and care aides did not perceive ACP as within their scope of practice, deferring to nurses.

